# Clinical Characteristics and Risk Factors for Cryptococcal Meningitis in Non-Acquired Immunodeficiency Syndrome Patients with Pulmonary Cryptococcosis: A 12-Year Hospital-Based Study

**DOI:** 10.3390/pathogens15060560

**Published:** 2026-05-22

**Authors:** Xiao Dang, Sha-Sha Wu, Lan Zhang, Li-Li Wu, Cai-Lin Guo, Wen Kang, Ye Zhang, Pei Li

**Affiliations:** 1 Department of Infectious Diseases, Tangdu Hospital, Fourth Military Medical University, Xi’an 710038, China; dangxiao86@163.com (X.D.); wss18991868503@163.com (S.-S.W.); 13572071374@163.com (L.Z.); kangwen1981@fmmu.edu.cn (W.K.); 2 Department of Infectious Diseases, Air Force Hospital of Southern Theatre Command, Guangzhou 510602, China; 13928776915@139.com; 3 Operation Room, Changchun Orthopedics Hospital, Changchun 130000, China; 15891705101@139.com

**Keywords:** *Cryptococcus*, cryptococcosis, pulmonary cryptococcosis, cryptococcal meningitis, clinical characteristics

## Abstract

In this study, we aimed to investigate the clinical characteristics of pulmonary cryptococcosis (PC) patients without acquired immunodeficiency disease (AIDS). In this retrospective study, a total of 101 patients with non-AIDS PC diagnosed at Tangdu Hospital between January 2014 and October 2025 were enrolled. The characteristics of demographic data, underlying diseases, clinical manifestations, laboratory indicators, and clinical outcomes were analyzed. Univariate and multivariate logistic regression analyses were used to identify risk factors for cryptococcal meningitis (CM). Among 101 patients (mean age 53.13 ± 12.31 years; 66.3% male), 56.4% were asymptomatic. Underlying diseases were present in 55.4% (mainly hypertension and diabetes), and CM occurred in 17.8% (18/101). Patients with CM had a higher proportion of underlying diseases (83.3% vs. 49.4%) and lower levels of red blood cells, hemoglobin, total protein, albumin, globulin, and potassium. Multivariate analysis revealed underlying diseases (OR = 7.246, 95% CI: 1.426~55.33), hypoglobulinemia (OR = 0.847, 95% CI: 0.734~0.956), and hypokalemia (OR = 0.177, 95% CI: 0.028~0.778) as independent risk factors for CM. The combined model showed good predictive value (AUC = 0.863). Non-AIDS PC often presents asymptomatically. Patients with underlying diseases, hypoglobulinemia, or hypokalemia are at significantly higher risk for concurrent CM and warrant aggressive central nervous system evaluation.

## 1. Introduction

Cryptococcal infection is a common invasive fungal disease that can affect various tissues and organs, including the lungs, central nervous system, skin, and bones. It predominantly occurs in patients with compromised cell-mediated immunity [1]. Risk factors for infection include human immunodeficiency virus (HIV) infection, malignancies, liver cirrhosis, diabetes mellitus, solid organ transplantation, autoimmune diseases, and the use of corticosteroids or biological agents [2,3,4]. Notably, cryptococcal infection can also occur in individuals without apparent immunosuppression [2]. Humans typically acquire the infection by inhaling aerosolized cryptococcal spores from the environment, initially causing pulmonary cryptococcosis (PC), or colonizing the respiratory tract and exhibiting pathogenicity when host immune function declines. Consequently, the clinical presentation of PC ranges from asymptomatic cases to severe acute respiratory distress syndrome [1]. Furthermore, *Cryptococcus* can disseminate from the lungs into the bloodstream and ultimately cross the blood–brain barrier to cause cryptococcal meningitis (CM) [1,5].

While cryptococcosis is a well-recognized opportunistic infection in patients with acquired immunodeficiency syndrome (AIDS), in recent years, we have witnessed a noticeable increase in the incidence of cryptococcal infections among non-AIDS populations [6]. A large 10-year multicenter study in China specifically highlighted the growing importance of understanding PC in patients with varying immune statuses, further emphasizing the need for research in this area [7]. The epidemiological shift has been reported globally. A Brazilian study of 94 cryptococcosis patients showed that only 30.9% were HIV-positive, while 41.5% were non-HIV immunosuppressed individuals and 20.2% were non-immunosuppressed or non-HIV hosts [5]. A large US population-based cohort study found that up to 90% of cryptococcosis cases occurred in non-HIV patients, including solid organ transplant recipients, patients with malignancies, and immunocompetent individuals [2]. Furthermore, a recent Australian and New Zealand multicenter study involving 46 hospitals found that 90% of 475 cryptococcosis cases occurred in HIV-negative patients, with emerging risk groups such as those with cancer and organ transplant recipients being prominently represented [8]. The World Health Organization has listed *Cryptococcus neoformans* as a priority fungal pathogen, with an estimated one million new cases and 600,000 deaths annually worldwide [9]. The global burden is further underscored by studies from regions like Northeastern Brazil, which report high mortality rates (46%) associated with HIV-related neurocryptococcosis, with common symptoms including fever (75%) and headache (62.5%) [10].

Despite this changing epidemiology, research specifically focusing on the clinical features and risk factors for dissemination in non-AIDS patients with PC remains limited. This knowledge gap may lead to insufficient awareness among clinicians, potentially resulting in misdiagnosis or delayed diagnosis. Therefore, this study retrospectively analyzes the clinical characteristics of 101 non-AIDS patients with PC treated at Tangdu Hospital between January 2014 and October 2025. The aim is to enhance diagnostic and therapeutic capabilities of clinicians for PC and ultimately reduce the mortality associated with this disease.

## 2. Materials and Methods

### 2.1. Study Population

**Figure 1:**
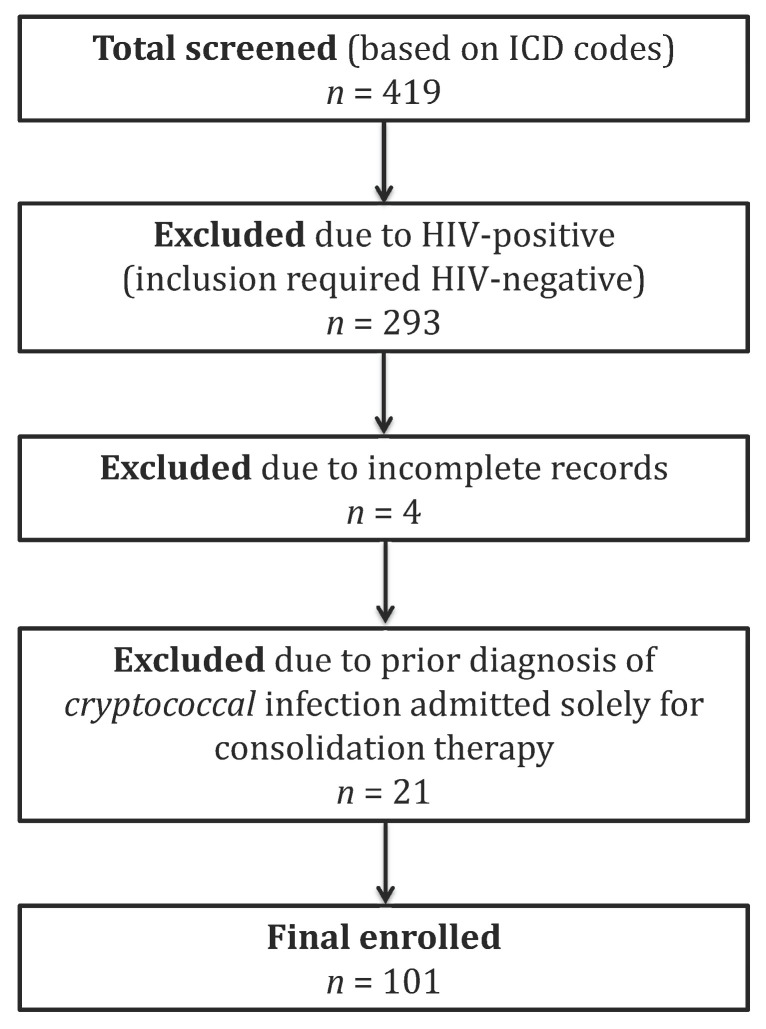
The flowchart for participant selection process.

All patients with cryptococcal infection diagnosed at Tangdu Hospital between January 2014 and October 2025 were consecutively screened for eligibility. Cases were identified through the hospital’s electronic medical record system using the International Classification of Diseases (ICD) codes for cryptococcosis (B45.0–B45.9). Both inpatient admission records and outpatient clinic records were reviewed. The participant selection process is summarized in Figure 1. Inclusion criteria were: (1) Negative for anti-HIV antibody test; (2) Definite diagnosis of cryptococcal infection based on at least one of the following: (a) histopathological examination of lung biopsy or surgical lung specimens with positive Grocott’s methenamine silver staining; (b) isolation of *Cryptococcus* from bronchoalveolar lavage fluid (BALF) culture; (c) detection of *Cryptococcus* by metagenomic next-generation sequencing (mNGS) of BALF. For patients with suspected central nervous system (CNS) involvement, diagnosis was confirmed by detection of *Cryptococcus* in cerebrospinal fluid (CSF) by mNGS or positive India ink stain. The mNGS procedure was performed as follows: 0.5 mL of CSF sample was centrifuged, and microbial DNA was extracted using the QIAamp Micro DNA Kit (Qiagen, Hilden, Germany). DNA libraries were prepared using the MGIEasy FS DNA Library Prep Kit and sequenced on the MGISEQ-2000 platform (MGI Tech, Shenzhen, China) with 50 bp single-end reads. Human host sequences were removed by mapping to the human reference genome, and remaining reads were aligned to the NCBI nucleotide database for microbial identification. A species was considered positive if it accounted for >0.01% of total mapped reads with at least 3 unique reads.

All enrolled patients had microbiological or histopathological confirmation of *Cryptococcus* infection. Exclusion criteria were: (1) incomplete clinical medical records; (2) patients previously diagnosed with cryptococcal infection and admitted solely for consolidation therapy or follow-up.

HIV-negative status was determined by a negative anti-HIV antibody test at hospital admission. Repeat HIV testing during follow-up was not routinely performed. We acknowledge that this approach cannot definitively exclude acute HIV infection during the serological window period; however, given the mean age of our cohort (53.13 years) and the clinical presentation (predominantly asymptomatic pulmonary nodules detected on routine health check-ups), the likelihood of undiagnosed acute HIV infection is extremely low.

CSF examination was not performed in all PC patients. Lumbar puncture was performed in patients presenting with neurological symptoms (including headache, altered consciousness, vomiting, or focal neurological deficits) or in those with neuroimaging findings suggestive of CNS involvement. Asymptomatic PC patients without neurological symptoms did not undergo CSF examination. This diagnostic strategy was based on clinical practice guidelines, which do not recommend routine lumbar puncture in asymptomatic PC patients, although we acknowledge that this approach may introduce selection bias.

Patients with documented coinfections (including bacterial pneumonia and chronic hepatitis B) were not excluded from the analysis, and these conditions were recorded as part of underlying diseases or concomitant infections. Patients with active tuberculosis, other invasive fungal infections, or other known immunosuppressive conditions (e.g., hematologic malignancies, solid organ transplantation) were not specifically excluded unless they met the exclusion criteria.

This study protocol was approved by the Ethics Committee of Tangdu Hospital (Approval number: K-HG-202601-15), and the requirement for informed consent was waived.

### 2.2. Study Design

Data collected from the medical records of each patient included: (1) demographic information (age, sex); (2) underlying diseases (hypertension, type 2 diabetes mellitus, malignant tumors, chronic hepatitis B, bacterial pneumonia, coronary artery disease, bronchial asthma, rheumatoid arthritis, cardiac arrhythmias, emphysema, hypothyroidism, and myasthenia gravis); (3) clinical manifestations (cough, headache, fever, chest tightness/shortness of breath, vomiting, altered consciousness, hemoptysis, or asymptomatic status); (4) laboratory findings—complete blood count [white blood cell (WBC) count, neutrophil count and percentage, lymphocyte percentage, monocyte percentage, platelet count, red blood cell count, hemoglobin], liver function tests [alanine aminotransferase (ALT), aspartate aminotransferase (AST), total bilirubin, total protein, albumin, globulin], renal function tests (blood urea nitrogen, creatinine, estimated glomerular filtration rate, uric acid), electrolytes (potassium, sodium, chloride, calcium), and blood glucose; (5) for patients with concurrent CM, CSF parameters (opening pressure, total cell count, leukocyte count, protein, glucose, chloride) and CSF diagnostic results (India ink staining, mNGS); (6) data on prior antifungal or antibacterial drug use within 30 days before admission were collected. Treatment regimens, including the type, dosage, and duration of antifungal therapy (fluconazole, itraconazole, voriconazole, amphotericin B, or combination therapy), as well as surgical interventions, were recorded; and (7) outcomes were categorized at hospital discharge as: recovery, death, and residual neurological sequelae (assessed at discharge for CM patients).

All laboratory parameters were collected at hospital admission. For the patients without prior antifungal therapy within 30 days, these parameters represent true baseline status. For the remaining patients with prior antifungal use, a sensitivity analysis was performed excluding these cases to assess potential confounding. All patients with suspected CNS involvement underwent neuroimaging (computed tomography or magnetic resonance imaging) prior to lumbar puncture. CSF examination was performed only in patients with neurological symptoms or abnormal neuroimaging findings. Follow-up data were limited to in-hospital outcomes. Patient status was assessed at hospital discharge (recovery or death). Long-term follow-up after discharge was not systematically performed.

The clinical characteristics of patients with PC, specifically focusing on those with concurrent CM, were analyzed. The clinical features between patients with PC who did and did not have concurrent CM were compared, and risk factors for developing CM in non-AIDS patients with PC were analyzed.

### 2.3. Statistical Analysis

Statistical analysis was performed using SPSS software version 25.0. Categorical data were presented as frequencies and constituent ratios, and comparisons between groups (patients without CM vs. patients with concurrent CM) were performed using the Chi-square test or Fisher’s exact test, as appropriate. Continuous data were first assessed for normality using the Shapiro–Wilk test. Normally distributed data were expressed as mean ± standard deviation ($\bar{x}$ ± *s*), and comparisons between two groups were performed using the independent samples *t*-test. Non-normally distributed data were expressed as median (interquartile range) [*M* (*Q1*, *Q3*)], and comparisons between two groups were performed using the Mann–Whitney *U* test. Variables with statistical differences in univariate analysis were included in the multivariate stepwise logistic regression analysis to identify independent risk factors for CM. In the univariate and multivariate analyses, laboratory parameters including total protein, albumin, globulin, and potassium were analyzed as continuous variables (using their measured values). The terms “hypoproteinemia”, “hypoalbuminemia”, “hypoglobulinemia”, and “hypokalemia” are used descriptively in the results to indicate significantly lower levels of these parameters in patients with concurrent CM compared to those without CM. Results were presented as odds ratios (OR) with 95% confidence intervals (CI). The predictive value of the multivariate model was assessed using receiver operating characteristic (ROC) curve analysis. A *p* value less than 0.05 was considered statistically significant.

## 3. Results

### 3.1. General Characteristics of Enrolled Patients

A total of 101 non-AIDS patients with cryptococcal infection admitted to Tangdu Hospital between January 2014 and October 2025 were enrolled. Among the 101 patients, 68 were male (67.3%) and 33 were female (32.7%), with a mean age of (53.13 ± 12.31) years. Fifty-seven patients (56.4%) were asymptomatic. The remaining 44 patients presented with various clinical manifestations, including cough (20.8%), headache (13.9%), fever (10.9%), chest tightness/shortness of breath (8.9%), vomiting (5.0%), altered consciousness (2.0%), and hemoptysis (1.0%). Underlying diseases were present in 56 patients (55.4%), comprising hypertension (24.8%), type 2 diabetes mellitus (22.8%), malignant tumors (9.9%), chronic hepatitis B (8.9%), bacterial pneumonia (6.9%), coronary artery disease (4.0%), bronchial asthma (4.0%), rheumatoid arthritis (3.0%), cardiac arrhythmias (3.0%), emphysema (2.0%), hypothyroidism (1.0%), and myasthenia gravis (1.0%). Of the total cohort, 83 patients did not have CM, while 18 patients had concurrent CM. One hundred patients recovered, and only one patient (with concurrent CM) died.

### 3.2. Clinical Characteristics of Asymptomatic Non-AIDS PC Patients

All 57 asymptomatic patients were among those without CM, including 38 males and 19 females, with a mean age of (53.75 ± 11.79) years. Underlying diseases were presented in 28 patients. All asymptomatic patients were diagnosed following the incidental discovery of pulmonary nodules on imaging during health check-ups. Nodule diameter was >8 mm or showed progressive enlargement on follow-up scans. All patients underwent surgical resection. Postoperative histopathology revealed chronic granulomatous inflammation of the lung tissue, with or without necrosis. Spherical yeast forms suggestive of *Cryptococcus* were observed locally in some patients. Grocott’s methenamine silver staining was positive in all tissue samples, confirming the diagnosis of cryptococcal infection. All patients recovered without mortality. The mean/median values of complete blood count, liver and kidney function tests, electrolytes, and blood glucose were within normal ranges (Table 1).

### 3.3. Comparison of Clinical Characteristics Between Non-AIDS PC Patients with and Without Concurrent CM

No statistically significant differences were observed in gender distribution or mean age between patients without and with concurrent CM (*p* > 0.05). The proportion of patients with underlying diseases was significantly higher in the patients with concurrent CM (83.3%) compared to those without (49.4%) (*p* = 0.018). Regarding hematological parameters, there were no significant differences between the two groups in WBC count, neutrophil count, lymphocyte percentage, monocyte percentage, or platelet count (*p* > 0.05). However, red blood cell count and hemoglobin levels were significantly lower in patients with concurrent CM (*p* < 0.001). For liver function indicators, no significant differences were found in ALT, AST, or total bilirubin levels (*p* > 0.05). Total protein, albumin, and globulin levels were significantly lower in the patients with concurrent CM (*p* < 0.01). There were no significant differences in renal function, uric acid, blood glucose, or sodium, chloride, and calcium levels between the two groups (*p* > 0.05). However, potassium levels were significantly lower in patients with concurrent CM (*p* = 0.004) (Table 2).

### 3.4. Clinical Characteristics of Non-AIDS PC Patients with Concurrent CM

Among the 18 patients with concurrent CM, 17 recovered and one died. CSF examination was performed in all patients. India ink staining was positive in 16 patients, while mNGS of CSF detected *Cryptococcus* in 2 patients. Initial CSF pressure was (188.7 ± 62.94) mmH_2_O, with elevated pressure (>200 mmH_2_O) observed in five patients (27.8%). CSF total cell count was 73.00 (13.00, 173.5) × 10^6^/L, and CSF leukocyte count was 64.00 (4.50, 138.0) × 10^6^/L, with elevated WBC (>100 × 10^6^/L) in 5 patients (27.8%). CSF protein was 591.8 (357.3, 1016) mg/L, with elevated protein (>300 mg/L) in 17 patients (94.4%). CSF glucose was 2.90 (2.04, 3.43) mmol/L, with decreased levels (less than half of concurrent blood glucose) in 14 patients (77.8%). CSF chloride was (123.2 ± 7.95) mmol/L, with decreased levels (<120 mmol/L) in 5 patients (27.8%).

### 3.5. Risk Factor Analysis for CM in Non-AIDS PC

**Figure 2:**
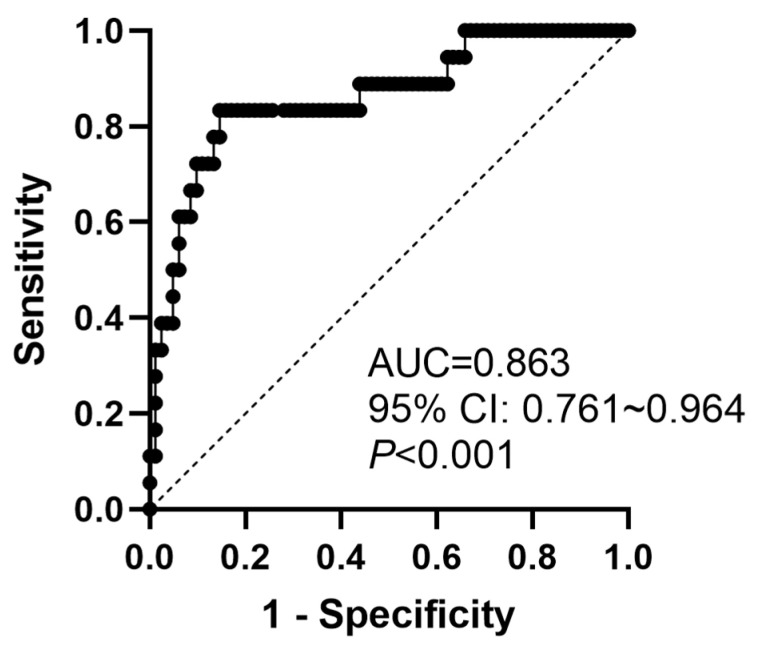
ROC curve of the multivariate model incorporating underlying diseases, globulin, and potassium for predicting cryptococcal meningitis.

Univariate analysis identified several factors associated with the development of CM in non-AIDS PC patients: presence of underlying diseases (OR = 5.122, 95% CI: 1.549~23.30), low platelet count (OR = 0.992, 95% CI: 0.984~0.999), low red blood cell count (OR = 0.192, 95% CI: 0.074–0.432), low hemoglobin (OR = 0.952, 95% CI: 0.925~0.976), hypoproteinemia (OR = 0.859, 95% CI: 0.789~0.923), hypoalbuminemia (OR = 0.852, 95% CI: 0.764~0.940), hypoglobulinemia (OR = 0.832, 95% CI: 0.733~0.929), and hypokalemia (OR = 0.187, 95% CI: 0.050~0.586) (Table 3). Further multivariate logistic regression analysis revealed that the presence of underlying diseases (OR = 7.246, 95% CI: 1.426~55.33), hypoglobulinemia (OR = 0.847, 95% CI: 0.734~0.956), and hypokalemia (OR = 0.177, 95% CI: 0.028~0.778) were independent risk factors for developing CM in non-AIDS patients with PC (Table 4). The ROC curve constructed by combining underlying diseases, globulin, and potassium levels is shown in Figure 2. The area under the curve was 0.863 (95% CI: 0.761~0.964).

### 3.6. Subgroup Analysis: Predictors of CM in Immunocompetent Patients

Among the 101 patients, 45 (44.6%) were classified as immunocompetent (no underlying diseases known to compromise immune function) and 56 (55.4%) as immunodeficient (presence of diabetes mellitus, malignancy, chronic hepatitis B, corticosteroid use, autoimmune disease, or other immunosuppressive conditions). In the immunocompetent subgroup (*n* = 45), 3 patients (6.7%) developed CM, compared to 15 patients (26.8%) in the immunodeficient subgroup (*p* = 0.008). Among immunocompetent patients, hypoglobulinemia remained a significant predictor of CM (OR = 0.812, 95% CI: 0.682~0.967, *p* = 0.019), while underlying diseases were by definition absent, and hypokalemia showed a trend toward association but did not reach statistical significance (OR = 0.153, 95% CI: 0.019~1.247, *p* = 0.079). The limited number of CM events in the immunocompetent group (*n* = 3) precluded multivariate analysis.

### 3.7. Treatment Regimens and In-Hospital Outcomes

Among the 18 CM patients, 17 (94.4%) received amphotericin B-based induction therapy (amphotericin B deoxycholate 0.7–1.0 mg/kg/day or liposomal amphotericin B 3–4 mg/kg/day) combined with fluconazole, followed by consolidation therapy with fluconazole (Table 5). One patient with mild CM and no neurological deficits was treated with fluconazole monotherapy. Among the 83 patients without CM, 57 (68.7%) underwent surgical resection alone (video-assisted thoracoscopic surgery or thoracotomy) with curative intent, and the remaining 26 patients received fluconazole monotherapy (400 mg daily) for 6–12 weeks (Table 5). Two patients (11.8%) among CM survivors had residual neurological sequelae (cognitive impairment and persistent headache) at discharge (Table 5).

### 3.8. Sensitivity Analysis Excluding Patients with Prior Antifungal Therapy

To exclude the potential confounding effects of prior antifungal treatment on laboratory parameters, we performed a sensitivity analysis after removing the 12 patients who had received antifungal or antibacterial therapy within 30 days before admission. In the remaining 89 patients, multivariate logistic regression confirmed that underlying diseases (OR = 7.112, 95% CI: 1.358~54.89), hypoglobulinemia (OR = 0.841, 95% CI: 0.725~0.962), and hypokalemia (OR = 0.169, 95% CI: 0.025~0.801) remained independent risk factors for CM, consistent with the primary analysis.

## 4. Discussion

This single-center retrospective study of 101 non-AIDS PC patients over 12 years revealed that over half (56.4%) were asymptomatic, over half (55.4%) had underlying diseases, and 17.8% had concurrent CM at diagnosis. The presence of underlying diseases, hypoglobulinemia, and hypokalemia were identified as independent risk factors for predicting CM. These findings have significant implications for early identification of high-risk patients and prevention of CNS dissemination.

The proportion of asymptomatic patients in our cohort (56.4%) was higher than the 30–40% reported in a recent Korean study of non-HIV cryptococcosis [6]. This discrepancy may be attributed to the widespread adoption of health check-ups and advances in imaging technology in China, leading to earlier detection of asymptomatic pulmonary nodules [11]. Laboratory parameters in these patients were generally within normal ranges, suggesting that in hosts without overt cellular immunodeficiency, *Cryptococcus* can form localized pulmonary lesions without eliciting systemic inflammatory responses [12]. However, it is noteworthy that even among asymptomatic patients, 49.1% had underlying diseases, suggesting that underlying immune dysregulation may be an important pathogenic background [2]. Therefore, incidentally discovered PC should not be simply regarded as benign colonization, especially in patients with underlying diseases who require assessment of dissemination risk [13].

Compared to AIDS patients, the clinical spectrum of non-AIDS PC shows significant heterogeneity [14]. Studies in HIV-positive patients with cryptococcosis consistently report headache (occurring in 58.6~62.5% of cases) and fever (54.3~75% of cases) as the predominant symptoms, along with high rates of elevated intracranial pressure (>200 mmH_2_O in 71.4%) and poor prognosis [1,10]. In contrast, AIDS patients often present with acute onset, prominent systemic symptoms, and CM rates often exceeding 50% [15], whereas non-AIDS patients predominantly present with asymptomatic or mild respiratory symptoms [4], with imaging findings mostly showing isolated nodules or localized consolidation [16]. Our data confirm this pattern. Regarding diagnosis, non-AIDS patients often require lung biopsy or surgical pathology for definitive diagnosis. Therapeutically, patients with localized disease have good prognoses with surgical resection or short-course antifungal therapy; however, once CM occurs, mortality increases significantly [17,18]. The mortality rate in our CM subgroup was 5.6% (1/18), consistent with literature reports.

The identification of underlying diseases, hypoglobulinemia, and hypokalemia as independent risk factors for CM in PC patients may collectively point to a central issue: compromised host immune defense. First, chronic underlying diseases (such as diabetes, liver cirrhosis, and malignancies) can impair innate and adaptive immunity through various mechanisms [19]. A recent study of hospitalized patients with cirrhosis found that *Cryptococcus neoformans* was the most common cause of meningoencephalitis (19%) and was associated with the highest mortality (33%), with ascites identified as an independent risk factor for cryptococcal meningitis (adjusted OR: 2.86) [3]. Second, hypoglobulinemia directly reflects humoral immune deficiency, and anti-capsular polysaccharide antibodies play a critical role in controlling hematogenous dissemination of *Cryptococcus* [20,21]. A large study of 525 PC patients that developed a nomogram for predicting CM found that immune parameters (including T cells, T helper cells, cytotoxic T cells, NK cells, and B cells) were significant predictors, reinforcing the central role of host immunity in preventing dissemination [22]. Third, hypokalemia is often associated with chronic wasting conditions and serves as an indicator of malnutrition and overall functional decline, potentially correlating with impaired immune cell function [23]. Therefore, patients with these characteristics should be considered at high risk for CM [9]. It is important to note that all laboratory parameters in this study were collected prior to antifungal treatment initiation, thereby excluding the possibility that the observed hypokalemia and hematologic abnormalities were iatrogenic effects of amphotericin B therapy, which is known to cause electrolyte disturbances and anemia during induction treatment. Nevertheless, future prospective studies with a treatment-naïve cohort are warranted to further validate these findings.

While immunosuppression is a well-recognized risk factor for disseminated cryptococcosis, our study provided several novel insights. First, hypoglobulinemia, which reflects humoral immune deficiency, was identified as an independent risk factor for CM (OR = 0.847, 95% CI: 0.734~0.956). Although anti-capsular polysaccharide antibodies play a critical role in controlling hematogenous dissemination of *Cryptococcus*, the specific contribution of humoral immunity has been relatively understudied compared to cellular immunity. Subgroup analysis revealed that even among immunocompetent patients (those without identifiable immunosuppressive conditions), hypoglobulinemia remained a significant predictor of CM. This finding suggests that subtle humoral immune abnormalities might predispose to CNS dissemination. Further studies with detailed immunological profiling are needed to elucidate the mechanisms underlying this observation. Second, hypokalemia at baseline (prior to antifungal therapy) emerged as an independent predictor of CM (OR = 0.177, 95% CI: 0.028~0.778), which might serve as a surrogate marker of chronic illness, malnutrition, and overall functional decline rather than a direct pathogenic factor. Third, our predictive model incorporating these three factors (underlying diseases, globulin, and potassium) demonstrated good discriminative ability (AUC = 0.863), providing a practical tool for clinical risk stratification.

This study has several limitations. First, the single-center retrospective design with the relatively small sample size may introduce selection bias and limit subgroup analyses. The proportion of asymptomatic patients may be overestimated. Second, molecular typing data for cryptococcal isolates were unavailable, preventing analysis of associations between different species or genotypes and clinical phenotypes [24]. Notably, a multicenter study from China characterizing *Cryptococcus gattii* isolates found that while VGI was the predominant genotype, VGII strains exhibited higher minimum inhibitory concentrations to antifungal agents, suggesting that genotype may influence drug susceptibility and clinical outcomes [25]. Furthermore, a study of HIV-positive patients in Beijing revealed that ST5 was the major sequence type (78.5%) among *Cryptococcus neoformans* isolates, indicating the importance of understanding local molecular epidemiology [26]. Third, dynamic monitoring of laboratory parameters during treatment was not performed because follow-up was not systematically performed according to a standardized protocol, and the duration of follow-up was variable. Long-term outcomes beyond hospital discharge were not uniformly documented. Future multicenter prospective studies integrating microbiological and immunological parameters are needed to more comprehensively elucidate the disease course and dissemination mechanisms in non-AIDS PC. In addition, this study lacked a control group of patients with non-cryptococcal meningitis (e.g., bacterial, viral, or tuberculous meningitis). Therefore, the identified risk predictors (underlying diseases, hypoglobulinemia, and hypokalemia) currently apply only to the prediction of concurrent CM in patients with known pulmonary cryptococcosis, and their specificity for cryptococcal *versus* other etiologies of meningitis has not been established. Caution is warranted when generalizing these findings to differential diagnosis between CM and other central nervous system infections.

## 5. Conclusions

In conclusion, non-AIDS PC patients often lack specific clinical manifestations, with a high proportion of asymptomatic cases. The presence of underlying diseases, hypoglobulinemia, and hypokalemia are key risk factors for predicting concurrent CM. For PC patients with these characteristics, active evaluation for possible CNS involvement is essential. Nevertheless, given the single-center design, the relatively small sample size, and the absence of a non-cryptococcal meningitis control group, these predictors should be interpreted with caution, and their validity must be confirmed in larger, multicenter cohorts before broad clinical application.

## Figures and Tables

**Table 1:** Clinical characteristics of asymptomatic non-AIDS patients with pulmonary cryptococcosis (*n* = 57).

Variable	Value
Gender (*n*, %)	
Male	38 (66.67%)
Female	19 (33.33%)
Age (years, $\bar{x}$ ± *s*)	53.75 ± 11.79
Underlying diseases (*n*, %)	
Yes	28 (49.12%)
No	29 (50.88%)
White blood cells [×10^9^/L, *M* (*Q1*, *Q3*)]	6.44 (5.24, 8.65)
Neutrophils [×10^9^/L, *M* (*Q1*, *Q3*)]	4.02 (2.93, 6.71)
Neutrophil percentage (%, $\bar{x}$ ± *s*)	67.89 ± 13.17
Lymphocyte percentage [%, *M* (*Q1*, *Q3*)]	22.80 (11.85, 31.10)
Monocyte percentage [%, *M* (*Q1*, *Q3*)]	6.50 (5.70, 8.15)
Platelets [×10^9^/L, *M* (*Q1*, *Q3*)]	200.0 (166.0, 243.5)
Red blood cells (×10^12^/L, $\bar{x}$ ± *s*)	4.48 ± 0.61
Hemoglobin (g/L, $\bar{x}$ ± *s*)	136.2 ± 20.00
ALT [IU/L, *M* (*Q1*, *Q3*)]	19.00 (13.00, 24.00)
AST [IU/L, *M* (*Q1*, *Q3*)]	21.00 (18.00, 25.00)
Total bilirubin [μmol/L, *M* (*Q1*, *Q3*)]	16.60 (12.40, 21.85)
Total protein (g/L, $\bar{x}$ ± *s*)	69.40 ± 7.35
Albumin (g/L, $\bar{x}$ ± *s*)	43.14 ± 5.22
Globulin (g/L, $\bar{x}$ ± *s*)	25.82 ± 5.30
Blood urea nitrogen [mmol/L, *M* (*Q1*, *Q3*))]	5.15 (4.10, 6.31)
Creatinine [μmol/L, *M* (*Q1*, *Q3*)]	65.0 (56.33, 75.85)
eGFR (mL/min/1.73 m^2^, $\bar{x}$ ± *s*)	99.59 ± 13.73
Uric acid (μmol/L, $\bar{x}$ ± *s*)	291.9 ± 83.02
K^+^ (mmol/L, $\bar{x}$ ± *s*)	4.17 ± 0.42
Na^+^ (mmol/L, $\bar{x}$ ± *s*)	140.2 ± 2.17
Cl^−^ (mmol/L, $\bar{x}$ ± *s*)	105.5 ± 2.61
Ca^2+^ (mmol/L, $\bar{x}$ ± *s*)	2.33 ± 0.14
Blood glucose [mmol/L, *M* (*Q1*, *Q3*)]	5.93 (5.31, 6.60)

ALT: alanine aminotransferase: alanine aminotransferase; AST: aspartate aminotransferase; eGFR: estimated glomerular filtration rate.

**Table 2:** Comparison of clinical characteristics between non-AIDS PC patients without and with concurrent CM.

Variable	Without CM (*n* = 83)	with CM (*n* = 18)	*p* Value
Gender (*n*, %)			0.732
Male	57 (68.67%)	11 (61.11%)	
Female	26 (31.33%)	7 (38.89%)	
Age (years, $\bar{x}$ ± *s*)	53.69 ± 12.28	50.56 ± 12.45	0.330
Underlying diseases (*n*, %)			0.018
Yes	41 (49.39%)	15 (83.33%)	
No	42 (50.61%)	3 (16.67%)	
White blood cells [×10^9^/L, *M* (*Q1*, *Q3*)]	6.41 (5.26, 8.46)	7.73 (4.93, 10.34)	0.572
Neutrophils [×10^9^/L, *M* (*Q1*, *Q3*)]	4.21 (3.20, 6.68)	5.75 (2.33, 8.43)	0.554
Neutrophil percentage (%,$\bar{x}$ ± *s*)	68.43 ± 12.34	69.57 ± 17.42	0.743
Lymphocyte percentage [%, *M* (*Q1*, *Q3*)]	21.30 (12.10, 30.60)	19.85(11.58, 27.50)	0.404
Monocyte percentage [%, *M* (*Q1*, *Q3*)]	6.70 (5.90, 8.50)	8.20 (5.95, 10.30)	0.137
Platelets [×10^9^/L, *M* (*Q1*, *Q3*)]	201.0 (165.0, 248.0)	158.5 (81.25, 271.0)	0.109
Red blood cells (×10^12^/L, $\bar{x}$ ± *s*)	4.49 ± 0.63	3.72 ± 0.73	<0.001
Hemoglobin (g/L, $\bar{x}$ ± *s*)	136.7 ± 20.48	113.1 ± 21.26	<0.001
ALT [IU/L, *M* (*Q1*, *Q3*)]	22.00 (14.00, 29.00)	21.50 (14.25, 38.25)	0.652
AST [IU/L, *M* (*Q1*, *Q3*)]	22.00 (18.00, 27.00)	19.00 (13.00, 26.75)	0.171
Total bilirubin [μmol/L, *M* (*Q1*, *Q3*)]	16.30 (11.72, 22.00)	15.37 (12.25, 21.38)	0.876
Total protein (g/L, $\bar{x}$ ± *s*)	70.70 ± 7.59	61.41 ± 7.79	<0.001
Albumin (g/L, $\bar{x}$ ± *s*)	43.03 ± 5.04	38.47 ± 5.62	<0.001
Globulin (g/L, $\bar{x}$ ± *s*)	27.74 ± 5.39	22.94 ± 6.91	0.002
Blood urea nitrogen [mmol/L, *M* (*Q1*, *Q3*)]	5.15 (4.10, 6.24)	4.97 (4.25, 7.59)	0.634
Creatinine [μmol/L, *M* (*Q1*, *Q3*))]	67.00 (56.00, 75.30)	60.50 (45.75, 74.90)	0.346
eGFR (mL/min/1.73 m^2^, $\bar{x}$ ± *s*)	100.7 ± 14.07	103.5 ± 27.96	0.542
Uric acid (μmol/L, $\bar{x}$ ± *s*)	298.8 ± 87.23	262.3 ± 88.83	0.112
K^+^ (mmol/L, $\bar{x}$ ± *s*)	4.06 ± 0.43	3.69 ± 0.62	0.004
Na^+^ (mmol/L, $\bar{x}$ ± *s*)	140.4 ± 2.49	139.6 ± 6.91	0.417
Cl^−^ (mmol/L, $\bar{x}$ ± *s*)	105.2 ± 3.00	104.0 ± 5.18	0.210
Ca^2+^ (mmol/L, $\bar{x}$ ± *s*)	2.32 ± 0.16	2.25 ± 0.16	0.104
Blood glucose [mmol/L, *M* (*Q1*, *Q3*)]	6.02 (5.32, 6.76)	5.66 (4.76, 7.92)	0.553

CM: cryptococcal meningitis; ALT: alanine aminotransferase; AST: aspartate aminotransferase; eGFR: estimated glomerular filtration rate.

**Table 3:** Univariate analysis of risk factors for cryptococcal meningitis in non-AIDS pulmonary cryptococcosis.

Variable	Odds Ratio	95% CI	*p* Value
Male gender	1.395	0.467~3.963	0.536
Age	0.979	0.938~1.021	0.328
Presence of underlying diseases	5.122	1.549~23.30	0.015
White blood cells	1.054	0.876~1.256	0.563
Neutrophils	1.074	0.902~1.270	0.410
Neutrophil percentage	1.007	0.969~1.049	0.740
Lymphocyte percentage	0.978	0.929~1.024	0.354
Monocyte percentage	1.104	0.991~1.261	0.089
Platelets	0.992	0.984~0.999	0.040
Red blood cells	0.192	0.074~0.432	<0.001
Hemoglobin	0.952	0.925~0.976	<0.001
ALT	1.015	0.999~1.045	0.153
AST	0.999	0.958~1.025	0.959
Total bilirubin	1.030	0.974~1.090	0.266
Total protein	0.859	0.789~0.923	<0.001
Albumin	0.852	0.764~0.940	0.002
Globulin	0.832	0.733~0.929	0.002
Blood urea nitrogen	1.180	0.957~1.459	0.110
Creatinine	0.995	0.964~1.023	0.753
eGFR	1.010	0.980~1.042	0.538
Uric acid	0.995	0.988~1.001	0.115
K^+^	0.187	0.050~0.586	0.007
Na^+^	0.942	0.812~1.084	0.412
Cl^−^	0.916	0.795~1.056	0.213
Ca^2+^	0.082	0.004~1.827	0.109
Blood glucose	1.178	0.907~1.535	0.196

CI: confidence interval; ALT: alanine aminotransferase; AST: aspartate aminotransferase; eGFR: estimated glomerular filtration rate.

**Table 4:** Multivariate analysis of risk factors for cryptococcal meningitis in non-AIDS pulmonary cryptococcosis.

Variable	*β* Value	S*_β_*	Wald *χ*^2^	Odds Ratio	95% Confidence Interval	*p* Value
Presence of underlying diseases	1.980	0.905	2.188	7.246	1.426~55.33	0.029
Platelets	−0.003	0.004	0.837	0.997	0.988~1.004	0.402
Red blood cells	−1.545	1.048	1.474	0.213	0.025~1.699	0.140
Hemoglobin	0.016	0.034	0.469	1.016	0.950~1.092	0.639
Albumin	−0.028	0.074	0.373	0.973	0.841~1.129	0.710
Globulin	−0.166	0.067	2.464	0.847	0.734~0.956	0.014
K^+^	−1.735	0.824	2.105	0.177	0.028~0.778	0.035

**Table 5:** Treatment regimens and in-hospital outcomes of non-AIDS pulmonary cryptococcosis with and without CM.

Parameter	Total (*n* = 101)	Without CM (*n* = 83)	with CM (*n* = 18)
Prior antifungal/antibacterial use within 30 days	12 (11.9%)	9 (10.8%)	3 (16.7%)
Antifungal regimen			
Fluconazole monotherapy	34 (33.7%)	32 (38.6%)	2 (11.1%)
Amphotericin B-based induction	18 (17.8%)	1 (1.2%)	17 (94.4%)
Combination therapy	6 (5.9%)	1 (1.2%)	5 (27.8%)
Surgical resection alone	57 (56.4%)	57 (68.7%)	0 (0%)
In-hospital outcomes at discharge			
Recovery	100 (99.0%)	83 (100%)	17 (94.4%)
Death	1 (1.0%)	0 (0%)	1 (5.6%)
Residual neurological sequelae at discharge (in CM survivors)	—	—	2 (11.8%)

CM: cryptococcal meningitis.

## Data Availability

The original contributions presented in the study are included in the article, and further inquiries can be directed to the corresponding authors.

## Abbreviations

The following abbreviations are used in this manuscript:

AIDS	acquired immunodeficiency disease
ALT	alanine aminotransferase
AST	aspartate aminotransferase
BALF	bronchoalveolar lavage fluid
CI	confidence intervals
CM	cryptococcal meningitis
CNS	central nervous system
CSF	cerebrospinal fluid
eGFR	estimated glomerular filtration rate
HIV	Three letter acronym
mNGS	metagenomic next-generation sequencing
OR	odds ratio
PC	pulmonary cryptococcosis
ROC	receiver operating characteristic
WBC	white blood cell
